# Evolutionary Relationships of Omani *Macrotermes subhyalinus*, *Macrotermitinae*

**DOI:** 10.3390/insects15090648

**Published:** 2024-08-29

**Authors:** Hilal S. AlShamakhi, Abdullah M. Al-Sadi, Lyn G. Cook

**Affiliations:** 1 Royal Court Affairs, Royal Estates Affairs, Muscat 113, Oman; hilal.alshamakhi@uqconnect.edu.au; 2 Department of Plant Sciences, College of Agricultural and Marine Sciences, Sultan Qaboos University, Al Khoud 123, Oman; 3 College of Agriculture, University of Al Dhaid, Sharjah P.O. Box 27272, United Arab Emirates; 4 School of Biological Sciences, The University of Queensland, Brisbane, QLD 4072, Australia; l.cook@uq.edu.au

**Keywords:** *M. subhyalinus*, *COI*, *28S*, Oman, pseudogene

## Abstract

**Simple Summary:**

In our recent investigation, we delved into the evolutionary relationships of *Macrotermes subhyalinus* populations in Oman. Our study employed genetic markers: the mitochondrial *COI* gene and the nuclear large-subunit ribosomal RNA (LSU rRNA *S28*). Our analyses revealed that *M. subhyalinus* in Oman belongs to an East African clade, distinct from its West African counterparts. This geographic divergence hints at separate evolutionary trajectories. Notably, we detected base composition bias among taxa in the *COI* gene. Our results support the idea that *Macrotermes* species might have dispersed out of Africa. Additionally, we report pseudogene copies of *28S* in *M. subhyalinus*, adding a novel dimension to termite genomics.

**Abstract:**

A study was conducted to investigate the evolutionary relationships of *Macrotermes subhyalinus* from Oman, in the southeastern part of the Arabian Peninsula. Sequences of the mitochondrial *COI* and the nuclear large-subunit ribosomal RNA (LSU rRNA, *28S*) genes were used to investigate the populations of *M. subhyalinus* across their distribution in Oman to determine their relationships with other *Macrotermes* species. Our findings indicate that *M. subhyalinus* in Oman is a member of an East African clade, distinct from those in West Africa. Analyses of the *COI* showed that there is base composition bias among the taxa (non-stationarity) that has not been considered in earlier studies. We provide the first report of pseudogene copies of *28S* in *M. subhyalinus* that are differentially amplified.

## 1. Introduction

Fungus-gardening termites (Termitidae: Macrotermitinae) have obligate symbioses with the fungus *Termitomyces* (Basidiomycete: Agaricales: Lyophyllaceae), and no free-living state of either partner has been recorded [1]. The fungus is gardened inside the nest in constant environmental conditions and provides the major food source for the host termite [2].

There appears to have been a single evolution of the fungus-gardening habit in the common ancestor of Macrotermitinae [3], which has been estimated to date between 19 and 19 and 49 million years ago (Ma) [4] or 30–108 Ma [5]. It has been argued that the fungus-gardening habit evolved in a forest biome, based on ancestral state reconstructions [6], the warm moist conditions needed by the fungus, and the molecular dating placing the crown of Macrotermitinae before the origins of savannah.

Morphological and molecular analyses indicate that Macrotermitinae are monophyletic [7,8,9,10]. They are distributed across the Palaeotropics and have the highest diversity of genera in Africa [9,11], leading to the common assumption that the subfamily is of African origin [11,12,13]. Overall, the fossil record and biogeographic patterns indicate that diversification during the Cenozoic and dispersal explain the current distributions [14]. Three genera are widely distributed in the Afrotropical and Oriental regions: *Macrotermes, Odontotermes*, and *Microtermes* [11]. The subfamily does not appear to disperse across significant water gaps and has not been reported in oceanic islands [11]. Unlike the Afrotropical genera *Odontotermes* and *Microtermes*, *Macrotermes* is more widely distributed across Oriental and Afrotropical regions [9].

There are well-known taxonomic problems within the genus *Macrotermes* [5,15,16,17], partly because there are few characteristics that differentiate between the currently recognized species [15], and there are problems in assigning newly collected material to described species [18]. One of the major problems is the non-monophyly of the African species *Macrotermes subhyalinus* [5,19,20], which replaced *M. bellicosus* after the split of *Macrotermes natalensis* [21] into *M. bellicosus* and *M. natalensis* [15]. The identification and differentiation of *M. subhyalinus* from other closely related termites have traditionally been based on the morphological features of all castes [15]. However, it is not always possible to differentiate and identify *M. subhyalinus* from other termites based on the available morphological descriptions because there are no synapomorphies for the species. For instance, *M. falciger* is commonly misidentified as *M. subhyalinus* due to morphological variations in both species across areas in which they both occur in Africa (e.g., Kenya and Tanzania) [22] (Table 1).

DNA sequence data, in particular, the mitochondrial (mt) gene cytochrome oxidase subunit 1 (*COI*), have begun to be used to identify and differentiate between species of *Macrotermes* [19]. Using overlapping datasets of mostly the same *COI* sequence data [5,19], found that populations of *M. subhyalinus* from West and East Africa did not form a monophyletic group: The West African samples clustered with *M. herus* and the East African samples clustered with *M. jeanneli*. Differences between Western and Eastern African lineages are also evident biologically, with East African colonies (e.g., in Kenya) having many open ventilation chimneys [19,23], whereas mounds in West Africa (e.g., Ivory Coast) have only a single open ventilation chimney [5,23]. The appearance of West African mounds of *M. subhyalinus* is very similar to the mounds of *M. herus* [24], in line with the sister relationship recovered from the analysis of *COI* [5]. The similarity in the mound structure and *COI* data casts doubt on the separate species status of *M. herus* and the West African populations of *M. subhyalinus* [5,19].

Here, we sampled populations of *M. subhyalinus* from across its distribution in Oman, on the Arabian Peninsula, to determine their relationships with other *Macrotermes*. We used *COI* data from the sampled populations and all other available Macrotermes *COI* sequences to assess the idea that *Macrotermes* have dispersed to the Arabian Peninsula out of Africa.

## 2. Materials and Methods

### 2.1. Specimen Sampling

**Figure 1:**
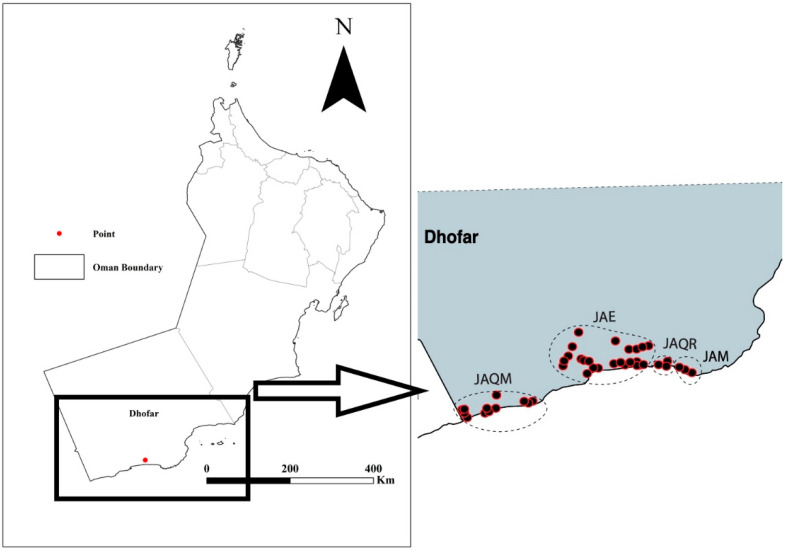
Map showing sampled localities (red dots) across Dhofar region in southern Oman: (JAQM) Jabal Qamar, (JAE) Jabal Eitin, (JAQR) Jabal Qara, and (JAM) Jabal Murbat (see Table S1).

Individuals of *M. subhyalinus* were collected from 76 colonies across Dhofar, southern Oman, during June and July 2015 (Figure 1). The initial processing of samples was performed within 12 h. Major soldiers, minor soldiers, major workers, minor workers, and imagoes were separated from fungal combs and surface-sterilized in 70% ethanol and then preserved in 99% ethanol until the extraction of genomic DNA.

### 2.2. DNA Extraction

#### 2.2.1. *COI*

One major soldier was selected from each colony. Genomic DNA was extracted from the head and thorax to reduce contamination from food sources and symbiotic microbes in the gut and abdomen. The samples were ground manually in 600 µL of 2X CTAB buffer and 10 µL of proteinase K (>600 mAU/mL). The mixture was then incubated on a hot plate for 2 h at 55 °C. The proteinase K was then denatured at 95 °C for 10 min. For RNA digestion, 0.5 µL of 100 mg/µL RNAse A was added to the mixture and incubated on the hot plate for 10 min at 37 °C. The subsequent steps were carried out as described previously [15].

#### 2.2.2. *28S*

For the autosomal large-subunit ribosomal RNA gene (*28S*), we amplified two regions, the expansion regions D3–D5 and D2–D3 of the rRNA. The former region was amplified with an annealing temperature of 50 °C using primers (Hux and Win) [25]. The D2–D3 region was amplified with an annealing temperature of 55 °C using the primers S3660 [26] and A335 [27]. The PCR consisted of an initial denaturing step for 2 min at 94 °C, then 40 cycles of denaturing at 94 °C (60 s), and annealing (60 s) and extension at 72 °C (120 s), followed by 10 min of additional extension at 72 °C.

### 2.3. Amplification and Sequencing Protocol

We amplified part of the mitochondrial gene cytochrome oxidase subunit 1 (*COI*) and the D3–D5 expansion regions of the nuclear large-subunit ribosomal RNA gene (LSU rRNA, *28S*) About 710 bp of *COI* was amplified with the Vrijenhoek [28] primers LCO-1490 (GGTCAACAAATCATAAAGATATTGG) and HCO-2198 (TAAACTTCAGGGTGACCAAAAAATCA) and a PCR consisting of an initial denaturing step for 2 min at 95 °C, and then 5 cycles of denaturing at 94 °C (40 s), annealing at 45 °C (40 s), and extension at 72 °C (70 s), followed by 40 cycles of denaturing at 94 °C (40 s), annealing at 51 °C (40 s), extension at 72 °C (70 s), and 5 min of additional extension at 72 °C. The D3–D5 region of *28S* was amplified with the primers Hux (ACACGGACCAAGGAGTCTAAC) and Win (GTCCTGCTGTCTTAAGCAACC) [25] at an annealing temperature of 50 °C. A subset of specimens was later sequenced for the D2–D3 region of *28S* using the primers S3660 (GAGAGTTMAASAGTACGTGAAAC) [26] and A335 (TCGGARGGAACCAGCTACTA) [29] at an annealing temperature of 55 °C. The PCR products were sequenced at Macrogen Inc. (Seoul, Korea) using BigDye 3.1 (Applied Biosystems, Foster city, CA, USA) and the Sanger method using the same primers used for the initial amplification.

### 2.4. Phylogenetic Analysis

*COI*

The samples were sequenced in both the forward and reverse directions and then edited and aligned using multiple-sequence alignment based on fast Fourier transform (MAFFT v7.017) [30] under the L-INS-I option (iterative refinement method incorporating local pairwise alignments), with a gap opening penalty = 1.5, 200 PAM/K = 2 scoring matrix, and offset value = 0.123, implemented in GENEIOUS R9 [31]. The same program was also used to estimate the *COI* divergence among regions using the max divergence option.

For the analysis of *COI*, 91 terminals (branches that represent sequences or hypothetical sequences at various points in evolutionary history) of the fungus-gardening species subfamily Macrotermitinae, representing 34 species and 7 genera, were included from GenBank (Table S2) along with 28 terminals representing 21 non-fungus-gardening species of Termitidae outside the subfamily Macrotermitinae to use as outgroups (Table S3). The non-fungus-gardening species *Sphaerotermes sphaerothorax* was also included because it has been placed in Macrotermitinae by some authors [3,9]. Some sequences were excluded due to ambiguity or that they are identical to other sequences (Table S4).

The *COI* gene alignment was checked for stop codons and indels in multiples other than three (the region is protein-coding). The first-, second-, and third-codon positions of *COI* were tested for deviations from stationary (base composition bias among taxa) using PAUP v4.0a146 [32] because this violates the assumptions of most phylogenetic methods. Two datasets were analyzed for *COI*: one comprising a wide selection of Termitidae and another comprising only Macrotermitinae. Third-codon positions of *COI* were excluded in sequences from across Termitidae datasets where there was non-stationarity. RNAfold [33] was used to estimate the secondary structure of several stem–loops in the *28S* RNA.

Distance-based dendrograms were calculated, and phylogenetic analyses were performed using two methods (maximum parsimony and Bayesian inference) [34,35], as explained previously [10].

JMODELTEST v2.1.6 [36] was used to select the model of sequence evolution to be applied to Bayesian analyses. The best model under the BIC criterion (HKY [37], including a proportion of invariable sites (I) and gamma-distributed among-site rate variation (Γ)) was then implemented as a prior in MrBayes v3.2.6 [38] and BEAST. MrBayes was run for 10 million generations, sampling every 1000 generations. Convergence was determined to have occurred when the differences in the harmonic mean of the two runs were less than two, and ESS values were indicated to be well-sampled using the software TRACER v1.6 [39]. The samples saved post-burn-in were pooled before calculating the majority rule consensus. Posterior probabilities of 0.95 and above were considered significant (for a comparison of bootstrap values and posterior probabilities.

*28S*

Alleles (variants of the sequence of nucleotides at a particular location, or locus, on a DNA molecule) of *28S* from heterozygotes were phased using PHASE v2.1.1 [40] and implemented in DnaSP v5.10 [41]. Highly ambiguous (multiple polymorphisms that were not confidently phased) and identical sequences were removed before the analysis to decrease polytomies that result from conflict and to reduce the over-representation of identical sequences to the model estimation. The sequences were aligned using MAFFT under the option (L-INS-I) implemented in GENEIOUS.

The whole of *28S* was tested for deviations from stationary using PAUP v4.0a146 [32] because this violates the assumptions of most phylogenetic methods. Sequences edited and aligned using MAFFT v7.017 [30] under the option (E-INS-I: iterative refinement method incorporating local alignment with generalized affine gap costs (gap opening penalty = 1.5; 200 PAM/K = 2 scoring matrix for nucleotide sequences) implemented in the Geneious program R9 [31]. The sequences were checked for base variations and site heterozygosity. In cases where heterozygosity is present, the PCR sequence product does not allow the determination of the phase of any variation sites detected or haplotypes, except in cases of homozygous samples or in cases in which all but one variation site is homozygous. Thus, computational inferences using probabilistic models are necessary to obtain haplotypes before running any phylogenetic analysis.

The PHASE v2.1.1 package program [40], implemented with the DNA polymorphism data (DnaSP v5.10) software [41], was used to assign the most probable haplotype constitution of each *28S* heterozygous sequence. The database containing homologous and heterozygous sequences was loaded into the PHASE software v 2.1.1 [40]. Analysis was performed using six independent runs with different seed values; number of iterations = 100; thinning interval = 1; and burn-in iterations = 100. Haplotypes with > 0.9 (90%) probability were considered. We included 21 terminals of the fungus-gardening species subfamily Macrotermitinae as in-taxa, and 61 terminals of non-fungus-gardening species (outgroup) from GenBank to infer the phylogenetic placement of the Omani Macrotermes using both parsimony and Bayesian analysis.

We checked for recombination among alleles using the “detect recombination” option implemented in the Geneious program based on a dual multiple change-point model [42,43]. Analysis was performed using a random seeding number; a chain length of 1,100,000; a subsampling frequency of every 10,000 states; and a 10% burn-in. Furthermore, we predicted the secondary structure for homozygous haplotypes A1, K4, and C4 using the computer method for folding an RNA molecule that finds a conformation of minimum free energy (MFE) using the values of the stacking and destabilizing energies (RNAfold) [33] (web server: http://rna.tbi.univie.ac.at, accessed on 8 November 2017). We compared the helix length, loop structures, and base composition of the predicted structures with the structural elements of the expansion regions D3–D5 of the Arthropods [44].

To test the effect of base composition heterogeneity among taxa on divergence times, we compared our dataset (with outgroup) before and after the RY coding of the third-codon position. For the non-RY-coded dataset, we applied two different relaxed-clock models, the lognormal uncorrelated relaxed clock model (UCLN) and the exponential uncorrelated relaxed clock model (UCED) implemented in BEAST using the HKY + I + G substitution model for the (1st + 2nd)- and 3rd-codon partitions. For the RY coding, we followed the Harrison et al. [45] method, coding the third-codon positions as purines or pyrimidines. RY coding reduces the effect of differences in the nucleotide composition between species resulting from C-T differences (pyrimidine bias), or between A and G (purine bias) [46]. Appropriate substitution models of the sequence evolution for each partition were assigned according to JMODELTEST v2.1.6. These were HKY + I + G for the (1st + 2nd)-codon partition and F81 + I + G [47] for the RY-coded 3rd-codon partition.

## 3. Results

### 3.1. COI

Specimens from 72 of the 76 collection locations in Oman were successfully sequenced for *COI*. All were identical across the 538 bp after trimming, and, therefore, only one representative sample was used in further analyses. When sequences from across the whole of Termitidae were included, there was significant non-stationarity (base composition bias among taxa) at third-codon positions (*p* = 0.0003) but not at first- and second-codon positions (Table 2). There was no non-stationarity at any codon position within Macrotermitinae (Table 2) or Macrotermitinae plus the non-fungus-gardening genus *Sphaerotermes* (Table 2).

**Figure 2:**
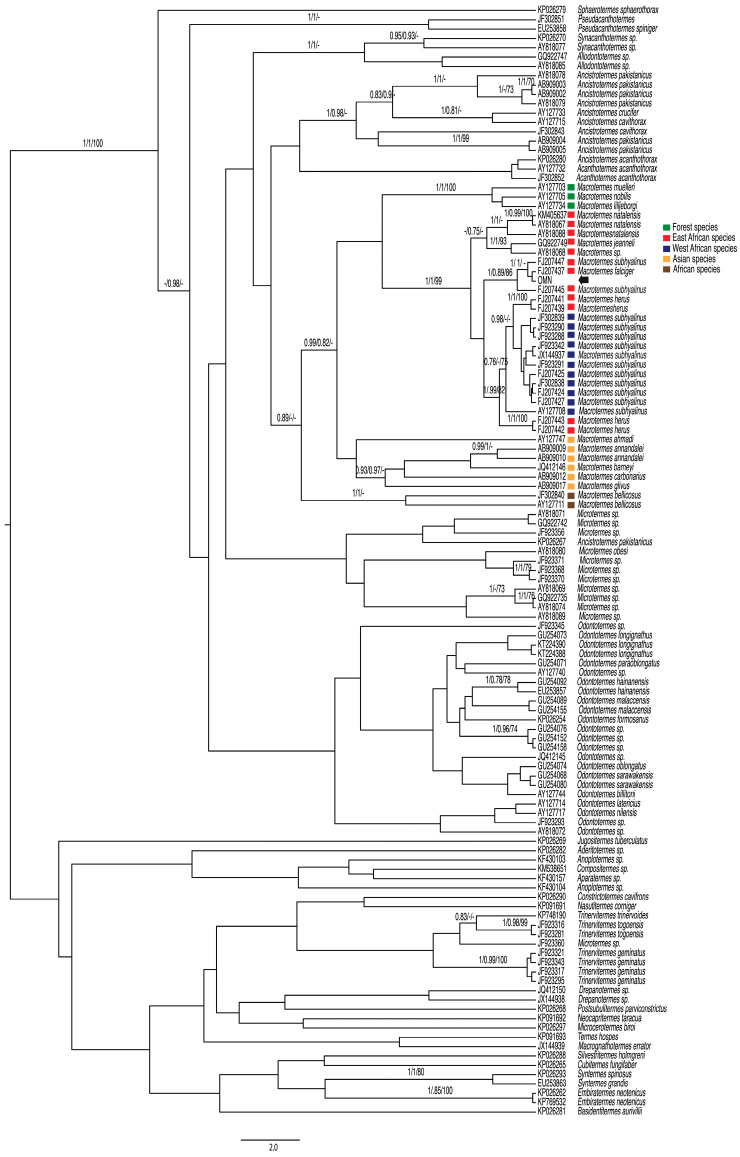
Phylogenetic relationships of *Macrotermes* from Oman and related fungus-gardening termites, including GenBank sequences. Phylogeny is from Beast Bayesian analysis of *COI* without RY coding, including all codon positions. Posterior probabilities (first and second numbers) and bootstrap support (third number) from 1000 pseudoreplicates are shown above internodes. Collection localities of *Macrotermes* are indicated by colored names (see insert legend). Scale bar: 2.0 bp. Arrow represents the Omani samples.

There was strong support from the analyses of *COI* without RY coding for the monophyly of Macrotermitinae plus *Sphaerotermes* (PP = 1.00 and BS = 100; Figure 2) but not for Macrotermitinae without *Sphaerotermes,* except in the MrBayes analysis (PP = 0.98; Figure 2). Similar results were obtained for *COI* based on the RY coding, with strong support from all analyses for the monophyly of Macrotermitinae plus *Sphaerotermes* (PP = 1.00; BS = 100) but not for Macrotermitinae without *Sphaerotermes*.

**Figure 3:**
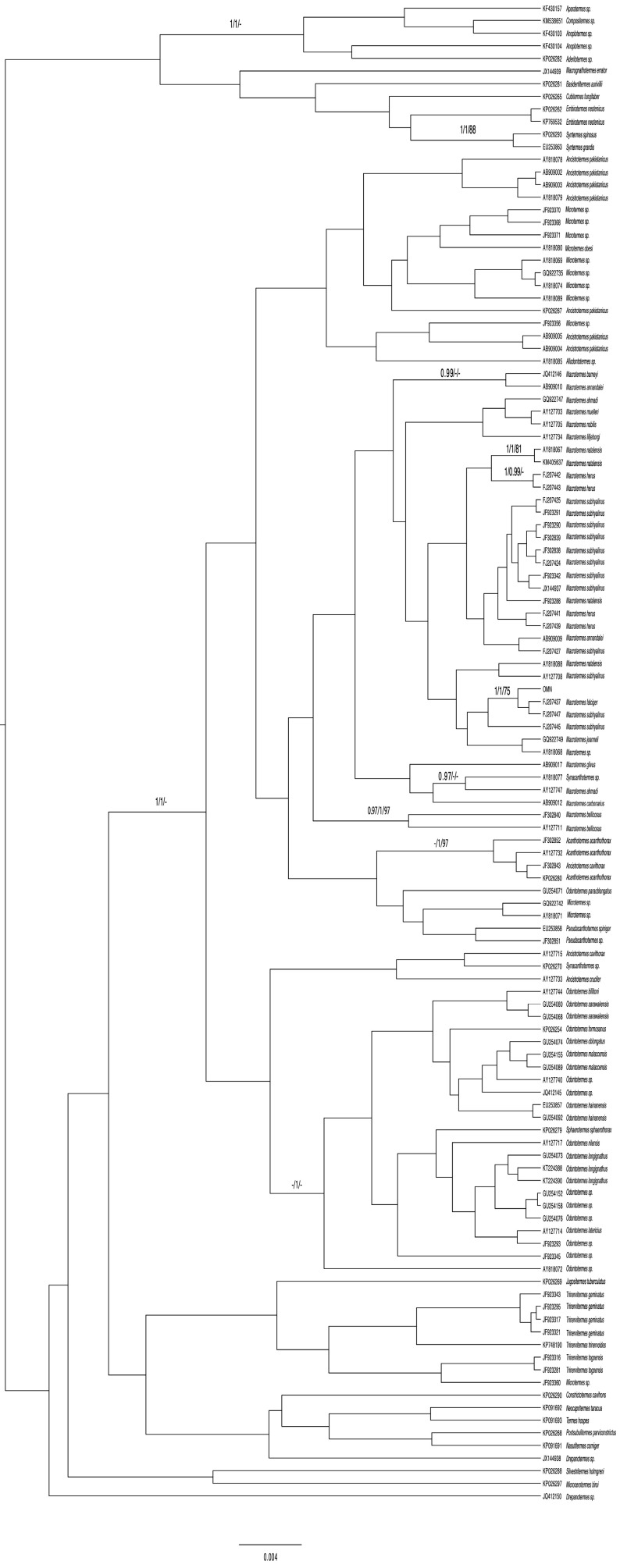
Phylogenetic relationships of *Macrotermes* from Oman and related fungus-gardening termites, including GenBank sequences. Phylogeny is from Beast Bayesian analysis of *COI* with RY coding, including all codon positions. Posterior probabilities (first and second numbers) and bootstrap support (third number) from 1000 pseudoreplicates are shown above internodes. Scale bar: 0.004 bp.

The tree topology and support values of the phylogeny estimates using RY coding were very different, with inaccurate grouping of lineages with similar base frequencies (e.g., Beast retrieved both genus Macrotermes and *Odontotermes* as non-monophyletic) (Figure 3) and posterior probabilities’ support values (e.g., the posterior probabilities for most groups were very low, at < 0.95, compared with the *COI* tree analysis without RY coding).

*Macrotermes* formed a monophyletic group in all analyses. but with little support (Figure 2). The three African forest species (*M. muelleri, M. nobilis,* and *M. lilljeborgi*) clustered together with strong support (PP = 1.00; BS = 100). The African species *M. natalensis, M. jeanneli, M. subhyalinus, M. falciger,* and *M. herus* formed a second well-supported clade (PP = 1.00; BS = 0.99) (Figure 2), whereas the Asian species (*M. ahmadi, M. annandalei, M. barneyi, M. glivus,* and *M. carbonarius*) formed a clade in the MrBayes analysis, but it was not well-supported (BS < 0.95). The two haplotypes of the African species *M. bellicosus* clustered together with strong support in the MP analyses but not in the Bayesian analysis (PP = 1.00; BS < 0.95).

*Macrotermes subhyalinus* was recovered as non-monophyletic in the analyses of *COI*. The haplotype presented in the Oman fell sister to a GenBank sequence recorded therein as being from *M. falciger* (Figure 2), with a 1.1% difference. Together, these formed a clade with sequences of *M. subhyalinus* from East Africa. The West African haplotypes from *M. subhyalinus* fell among sequences of *M. herus* with strong support in the MP analyses but not in the Bayesian analysis (PP = 1.00; BS < 0.95).

In contrast with the lack of variation in *COI* among Omani specimens, there was up to 0.5% divergence among the *M. subhyalinus* specimens from Kenya and up to 0.2% divergence in another *Macrotermes michaelseni* in Kenya.

### 3.2. 28S

**Figure 4:**
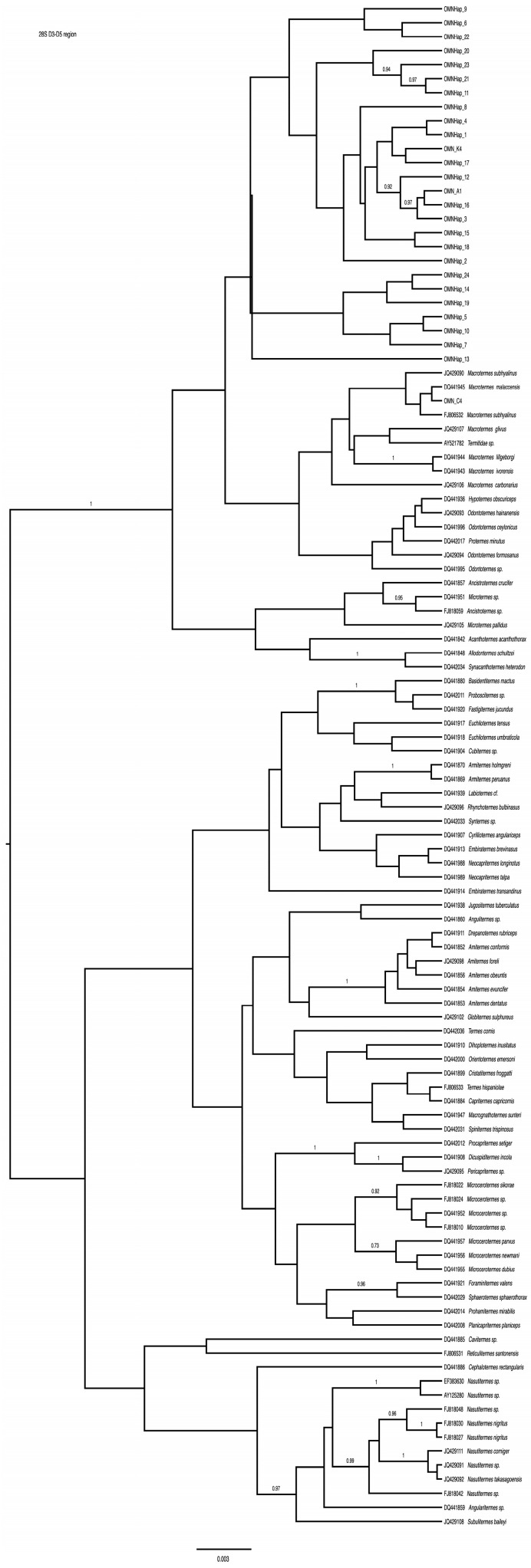
Phylogenetic placement of the Omani Macrotermes populations and its relationship to other related fungus-gardening termite, including Genbank specimens based on Bayesian analysis of 28S D3–D5 region of Arthropods. Posterior probabilities are shown above internodes. Scale bar: 0.003 bp.

We amplified multiple alleles of *28S* from 68 specimens collected in Oman. In total, 3 alleles were found in the homozygous state, but most individuals (*n* = 57) were heterozygous (an allele is represented by 1 or more base pair sequence differences for a given stem loop). One allele (C4) (Figure 4) was identical to those of *M. subhyalinus* (FJ806532) and *M. malaccensis* (DQ441945) in GenBank, whereas the other alleles fell outside GenBank accessions of *Macrotermes* and its sister genera but inside Macrotermitinae (Figure 4). Phasing estimated 24 haplotypes (probability > 0.9). The phylogenetic trees inferred from both the parsimony and Bayesian methods were highly similar. Only one homozygous specimen (haplotype C4) was placed within Macrotermes, and the rest formed a clade sister to the Macrotermitinae (Figure 1).

**Figure 5:**
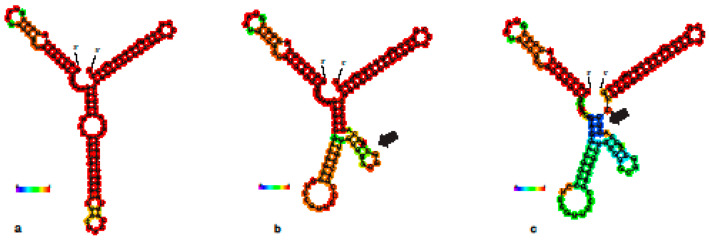
Minimum Free Energy (MFE) structure for the homozygous haplotype C4, drawing encoding base-pair probabilities. (**a**) C4 haplotype formed similar helix length and loops to the structural elements of Arthropods. (**b**) Minimum Free Energy (MFE) structure for the homozygous haplotype K4, drawing encoding base-pair probabilities, black arrow point to the changes in loops structure and variability in helix length compared to 2a. (**c**) Minimum Free Energy (MFE) structure for the homozygous haplotype A1, drawing encoding base-pair probabilities, black arrow point to the changes in loops structure and variability in helix length compared to 2a.

The recombination test detected no recombination rate among the tested haplotypes. The homozygous sequence (C4) formed similar helix lengths and loops to the structural elements of Arthropods (Figure 5a). In contrast, there was significant variability in the helix lengths, loop structures, sequence lengths, and base composition of the conserved regions in (K4, A1) (Figure 5b,c), which indicated the presence of multiple defunct relatives to the normal *28S* gene or pseudogenes. The estimation of the RNA secondary structure using RNAfold indicated that these copies were likely to be pseudogenes, defunct copies of *28S* that formed a different secondary structure for the D3 region than that estimated for all other arthropods. The amplification of the D2–D3 region of 28S using the primers S3660 and A335 recovered only the C4 variant from all the Omani specimens trialed, including those that were identified as heterozygous when using the primer pair from Hux and Win [34].

## 4. Discussion

The populations of *M. subhyalinus* across their distribution in Oman are members of an East African clade and distinct from *M. subhyalinus* in West Africa, and there was no genetic diversity found in Oman. Analyses of *COI* showed that there is base composition bias among the taxa. There is no specific evidence or documentation indicating that *M. falciger* has been introduced into Oman. The inclusion of *M. falciger* in the East African clade can be explained by geographic proximity. When populations are geographically closer, they are more likely to experience gene flow, which can lead to genetic similarities. This proximity facilitates interactions such as migration and interbreeding, which can result in the observed genetic relatedness. Therefore, the close geographic distance between *M. falciger* and East African *subhyalinus* populations likely contributed to their genetic similarities and their inclusion in the same clade. In addition, there are pseudogene copies of *28S* in *M. subhyalinus* that are differentially amplified with different primers. The inclusion of *COI* and *28S* from the same samples was crucial for this study. These genetic markers provided complementary information that was essential for accurately distinguishing between the East and West populations. The COI marker, being mitochondrial, offers insights into maternal lineage, while the *28S* marker, being nuclear, provides a broader genetic perspective. Together, they enhanced the resolution of our phylogenetic analysis, leading to the clear separation of the populations. Non-stationarity violates the assumptions of most methods of phylogenetic analysis, including MP, ML, and Bayesian methods [48], and potentially leads to the erroneous grouping of lineages with similar base frequencies. It might also artificially increase the observable differences between taxa and lead to the inappropriate modeling of substitution rates among bases (averaging such that parameters do not fit either bias), which may affect the molecular results through the perturbation of relative branch lengths.

Both genes analyzed here place the Omani populations of *Macrotermes* with *M. subhyalinus* from East Africa, but a sequence of *M. falciger* is also in the clade (Figure 2). The lack of genetic variation in *COI* among Omani populations, and the variation among Kenyan populations, indicates that *Macrotermes subhyalinus* likely dispersed into Oman. The lack of variation among populations could be a result of a relatively recent single establishment event or indicate a genetic bottleneck sometime after establishment (e.g., [49]). The lack of genetic diversity in *M. subhyalinus* suggests a bottleneck event, which could have reduced the population size and genetic variation. However, the close genetic relationship between *M. falciger* and East African *M. subhyalinus* raises questions about potential genetic exchange. The possibility of genetic exchange between these two groups lacks direct evidence. Further studies, such as more detailed genetic analyses or ecological investigations, could provide additional insights into the relationship between these populations and help clarify whether genetic exchange has occurred.

The relationship of Omani populations with *M. subhyalinus* and *M. falciger* from eastern Africa makes sense in terms of biogeography: Oman and the rest of the Arabian Peninsula are geographically closer to East Africa. More finely resolving markers and better sampling from Africa are needed to assess the biogeography of Omani *M. subhyalinus*.

The *COI* haplotype from Omani termites identified as *M. subhyalinus* fell sister to a haplotype from a termite identified as *M. falciger,* and samples identified as *M. subhyalinus* did not form a monophyletic group (Figure 2). There are well-known problems around the taxonomy of species of *Macrotermes* [5,15,16,19,50], and our analyses reiterate that *M. subhyalinus* is not monophyletic. To date, there has been an over-reliance on data from *COI* for estimating relationships among Macrotermitinae and other Termitidae. Furthermore, there is little overlap between the species included in studies using *COI* and those using *28S*, and, other than here, none have used the same specimens. Resolution of the species status of *M. subhyalinus* will need more sampling of multiple nuclear genes from a geographically comprehensive sampling of the population of *M. subhyalinus* and other closely related species, such as has been carried out with *Calligrapha* [51] and *Gonioctena* [52] (Coleoptera: Chrysomelidae).

The mounds of *M. subhyalinus* in Oman have many open ventilation chimneys, similar to those described for *M. subhyalinus* in Kenya [5,23], whereas the mounds in West Africa have only one large opening [23]. The relationship between the Omani populations of *M. subhyalinus* is, therefore, consistent with previous observations that the mound shape differs between the eastern and western clades of *M. subhyalinus*. This is further evidence that the fossil mound cannot be definitively assigned to an extant species and that the species differs between east and west and further supports the consideration of species status for the two types.

*Macrotermes subhyalinus* from Oman is closely related to *M. falciger* and *M. subhyalinus* from East Africa. The lack of diversity at *COI* and *28S* suggests that populations have only recently arrived in Oman from only a few founding individuals. Furthermore, our analyses reiterate that *M. subhyalinus* is not monophyletic, with the populations from West Africa having a different sister group from East African populations. More molecular data and broad geographic sampling are needed to resolve the taxonomy of *M. subhyalinus*.

## Figures and Tables

**Table 1:** Main overlap in characteristics between *M. falciger* and *M. subhyalinus* [15].

Major Soldier Head Capsule			Distinctive Features	Mound Structure	Swarming Behavior	Known Distribution
*M. falciger*	Measurements: 142 specimens from 64 localities, range is in millimeters Length (5.20–7.43) Width (4.00–6.14) Length of left mandible (2.85–4.01) Max. length of pronotum (1.70–261) Max. width of pronotum (3.30–5.18)	Colors: Dark chestnut-brown	Major soldiers of *M. falciger* are usually darker and are characterized by huge thoracic nota, a wide head, thick antennae, and conspicuous pilosity of the gula The minor soldiers have longer tibiae and more incurved mandibles than in *M. subhyalinus* Imagos of *M. falciger* are usually larger than those of *M. subhyalinus* and have proportionately smaller eyes and longer tibiae, and the depressed area around the fontanelle also differs in shape	Low hummocks, but can vary under different circumstances	Just after dark	Predominantly a woodland species, which is widely distributed in eastern and southern parts of Africa
*M. subhyalinus*	Measurements: 456 specimens from 163 localities, range is in millimeters Length (4.13–6.21) Width (3.26–4.89) Length of left mandible (2.52–3.14) Max. length of pronotum (1.43–1.90) Max. width of pronotum (2.55–3.74)	Red–yellow to reddish brown		Erects tall and spired, but can vary under different circumstances	Around midnight	Widely distributed through West, Central, and East Africa

**Table 2:** Base frequencies among taxa and chi-square test of homogeneity of state frequencies across taxa. Significant deviation from stationarity is in bold.

	Mean	A	C	G	T	Sites	Variable Sites	Among Taxa		
								Chi-Square	df	*p* > 0.05
*Macrotermitinae*	1st-codon position	0.32415	0.23234	0.27605	0.16746	179.00	42	24.909676	264	1.00000000
	2nd-codon position	0.13723	0.27883	0.17032	0.41362	178.99	15	3.245748	264	1.00000000
	3rd-codon position	0.48886	0.28916	0.07286	0.14912	178.99	168	249.222679	264	0.81496609
	All codon positions	0.31661	0.26682	0.17320	0.24337	536.97	225	78.074355	264	1.00000000
*Macrotermitinae* and *outgroup*	1st-codon position	0.32537	0.22856	0.27486	0.17121	179.00	49	53.197546	354	1.00000000
	2nd-codon position	0.13667	0.27773	0.17071	0.41488	178.99	17	4.698318	354	1.00000000
	3rd-codon position	0.48925	0.27340	0.06934	0.16801	178.99	169	518.419339	354	0.00000003
	All codon positions	0.31705	0.25988	0.17167	0.25140	536.98	235	194.923233	354	1.00000000
*Outgroup* only	1st-codon position	0.32880	0.21623	0.27132	0.18365	179.00	30	17.396548	87	1.00000000
	2nd-codon position	0.13501	0.27412	0.17188	0.41899	179.00	3	0.638419	87	1.00000000
	3rd-codon position	0.49300	0.22388	0.05703	0.22609	179.00	154	60.210541	87	0.98730157
	All codon positions	0.31880	0.23802	0.16683	0.27634	537.00	187	24.159910	87	1.00000000
RY coding: *Macrotermitinae* and *outgroup*	1st-codon position	0.32537	0.22856	0.27486	0.17121	178.97	49	53.196584	354	1.00000000
	2nd-codon position	0.13667	0.27773	0.17071	0.41488	178.98	17	4.698318	354	1.00000000
	3rd-codon position	0.27928	0.22072	0.27982	0.22072	178.98	104	38.248258	354	1.00000000
	All codon positions	0.24879	0.23697	0.23992	0.27432	536.94	170	47.366512	354	1.00000000

## Data Availability

The original data presented in this study are available in the Supplementary Materials.

## Supplementary Materials

The following supporting information can be downloaded at https://www.mdpi.com/article/10.3390/insects15090648/s1. Table S1: location longitude, latitude, and elevation of the mounds used in this study; Table S2: origin, and GenBank accession number of the fungus-gardening termite Macrotermitinae sequences used in this study. Table S3: Species of non-fungus-gardening Termitidae included as outgroup in this study. Table S4: GenBank accession numbers of sequences excluded in this study and reasons of exclusion.
