# Supplementary material for: Evolutionary Relationships of Omani Macrotermes subhyalinus, Macrotermitinae
Source: Insects. 2024 Aug 29;15(9):648. doi: 10.3390/insects15090648 (PMC11432140; doi:10.3390/insects15090648)
Supplement: Supplementary file 1 [file insects-15-00648-s001.zip › insects-2959991-supplementary.pdf]

Supplementary Materials

Table S1: location, longitude, latitude, and elevation of the mounds used in this study

Sr.	Mound No.	Longitude	Latitude	Elevation
1	**A01**	16°40'31.8"N	053°06'06.6"E	903m
2	**A02**	16°40'30.4"N	053°06'07.6"E	887m
3	**A03**	16°40'30.5"N	053°06'11.3"E	870m
4	**A04**	16°40'26.7"N	053°06'17.4"E	812m
5	**A05**	16°40'28.6"N	053°06'28.3"E	771m
6	**A06**	16°40'55.0"N	053°06'30.1"E	843m
7	**B01**	16°42'01.2"N	053°11'50.7"E	437m
8	**B02**	16°42'02.8"N	053°11'49.6"E	439m
9	**B03**	16°42'05.1"N	053°11'41.4"E	472m
10	**B04**	16°42'14.4"N	053°11'46.7"E	473m
11	**B05**	16°42'24.4"N	053°11'57.2"E	492m
12	**B06**	16°42'34.1"N	053°12'11.6"E	512m
13	**B07**	16°42'25.8"N	053°12'49.2"E	434m
14	**C01**	16°47'07.9"N	053°16'57.7"E	1038m
15	**C02**	16°47'03.6"N	053°16'56.5"E	1032m
16	**C03**	16°47'01.3"N	053°17'14.7"E	1036m
17	**C04**	16°46'36.0"N	053°17'36.9"E	1009m
18	**D01**	16°47'55.6"N	053°25'52.4"E	882m
19	**E01**	17°06'16.8"N	054°35'00.4"E	678m
20	**E02**	17°06'12.7"N	054°34'55.9"E	672m
21	**E03**	17.°06'09.0"N	054°34'53.7"E	668m
22	**E04**	17°06'13.4"N	054°34'45.5"E	664m
23	**E05**	17°06'13.1"N	054°35'19.5"E	688m

24	**F01**	17°04'24.2"N	054°36'30.2"E	711m
25	**F02**	17°04'25.9"N	054°36'31.7"E	710m
26	**F03**	17°04'18.8"N	054°36'34.9"E	689m
27	**F04**	17°04'06.9"N	054°36'04.8"E	597m
28	**G01**	17°03'14.4"N	054°33'11.3"E	482m
29	**G02**	17°03'26.3"N	054°33'21.7"E	449m
30	**G03**	17°03'31.5"N	054°33'06.2"E	469m
31	**H01**	17°05'36.2"N	054°22'58.1"E	559m
32	**H02**	17°05'37.9"N	054°22'58.2"E	561m
33	**H03**	17°05'41.4"N	054°22'48.4"E	575m
34	**H04**	17°05'41.7"N	054°22'34.7"E	557m
35	**I01**	17°04'07.7"N	054°24'12.5"E	91m
36	**J01**	17°11'31.8"N	054°23'54.0"E	800m
37	**J02**	17°11'39.8"N	054°23'56.5"E	812m
38	**K01**	17°09'36.0"N	054°10'11.9"E	801m
39	**K02**	17°09'31.8"N	054°03'08.1v	459m
40	**K03**	17°09'32.1"N	054°03'08.5"E	510m
41	**K04**	17°09'31.5"N	054°02'49.2"E	490m
42	**K05**	17°09'43.9"N	054°02'47.3"E	529m
43	**K06**	17°07'10.1"N	054°00'23.7"E	632m
44	**K07**	17°07'24.0"N	054°00'25.7"E	705m
45	**K08**	17°06'28.2"N	053°59'40.0"E	716m
46	**K09**	17°06'28.3"N	053°59'39.9"E	717m
47	**K10**	17°05'33.2"N	054°00'04.0"E	662m
48	**L01**	17°03'01.3"N	053°53'57.7"E	886m
49	**L02**	17°02'59.4"N	053°53'57.0"E	872m
50	**L03**	17°02'59.4"N	053°53'57.1"E	879m

51	**L04**	17°04'16.9"N	053°54'48.7"E	834m
52	**L05**	17°03'26.5"N	053°56'18.6"E	769m
53	**M01**	17°02'29.2"N	053°50'38.5"E	974m
54	**M02**	17°02'28.8"N	053°50'47.8"E	1001m
55	**M03**	17°02'25.3"N	053°50'55.5"E	1005m
56	**M04**	17°02'16.0"N	053°50'54.2"E	1030m
57	**M05**	17°02'08.5"N	053°51'05.1"E	1000m
58	**N01**	17°04'26.8"N	054°03'11.8"E	80m
59	**N02**	17°04'41.8"N	054°03'28.5"E	78m
60	**N03**	17°04'39.4"N	054°03'16.6"E	90m
61	**N04**	17°04'48.9"N	054°03'17.3"E	90m
62	**N05**	17°04'47.6"N	054°03'11.3"E	92m
63	**O01**	17°04'56.5"N	054°04'15.8"E	120m
64	**O02**	17°04'56.8"N	054°04'14.7"E	100m
65	**O03**	17°04'58.9"N	054°04'16.0"E	102m
66	**O04**	17°05'04.7"N	054°04'29.9"E	102m
67	**O05**	17°05'23.2"N	054°0'436.0"E	127m
68	**P01**	17°05'58.8"N	054°01'50.0"E	607m
69	**P02**	17°05'51.7"N	054°01'53.8"E	568m
70	**P03**	17°06'04.9"N	054°01'47.5"E	613m
71	**P04**	17°06'54.1"N	054°01'31.1"E	623m
72	**Q01**	17°02'55.0"N	054°00'13.5"E	113m
73	**Q02**	17°02'55.0"N	054°00'12.1"E	115m
74	**Q03**	17°03'01.5"N	054°00'09.1"E	120m
75	**Qo4**	17°03'07.1"N	054°00'04.7"E	132m
76	**Q05**	17°03'06.9"N	053°59'59.9"E	134m

Table S2 Name, origin, and GenBank accession number of the fungus-gardening termite Macrotermitinae sequences used in this study

Genus	Species	Continent	Location	Accession No.
Macrotermes	*falciger*	Africa	Kenya	FJ207437
Macrotermes	*subhyalinus*	Africa	Kenya	FJ207447
Macrotermes	*subhyalinus*	Africa	Kenya	FJ207445
Macrotermes	*jeanneli*	Africa	Kenya	GQ922749
Macrotermes	*Sp.*	Africa	South Africa	AY818068
Macrotermes	*natalensis*	Africa	South Africa	KM405637
Macrotermes	*natalensis*	Africa	South Africa	AY818067
Macrotermes	*natalensis*	Africa	South Africa	AY818088
Macrotermes	*subhyalinus*	Africa	-	JX144937
Macrotermes	*subhyalinus*	Africa	Ivory Coast	JF302838
Macrotermes	*subhyalinus*	Africa	Ivory Coast	JF302839
Macrotermes	*subhyalinus*	Africa	Ivory Coast	JF923290
Macrotermes	*subhyalinus*	Africa	Ivory Coast	JF923288
Macrotermes	*subhyalinus*	Africa	Ivory Coast	JF923291
Macrotermes	*subhyalinus*	Africa	Ivory Coast	JF923342
Macrotermes	*subhyalinus*	Africa	Ivory Coast	FJ207427

Macrotermes	*subhyalinus*	Africa	Ivory Coast	FJ207425
Macrotermes	*subhyalinus*	Africa	Ivory Coast	FJ207424
Macrotermes	*hersus*	Africa	Kenya	FJ207441
Macrotermes	*hersus*	Africa	Kenya	FJ207439
Macrotermes	*subhyalinus*	Africa	Senegal	AY127708
Macrotermes	*hersus*	Africa	Kenya	FJ207442
Macrotermes	*hersus*	Africa	Kenya	FJ207443
Macrotermes	*muelleri*	Africa	Gabon	AY127703
Macrotermes	*nobilis*	Africa	Gabon	AY127705
Macrotermes	*lilljeborgi*	Africa	Cameroon	AY127734
Macrotermes	*barneyi*	Asia	-	JQ412146
Macrotermes	*annandalei*	Asia	-	AB909010
Macrotermes	*annandalei*	Asia	-	AB909009
Macrotermes	*glivus*	Asia	-	AB909017
Macrotermes	*bellicosus*	Africa	Senegal	AY127711
Macrotermes	*bellicosus*	Africa	Ivory Coast	JF302840
Macrotermes	*ahmadi*	Asia	Indonesia	AY127747
Macrotermes	*carbonarius*	Africa	Ivory Coast	AB909012

Table S3 Species of non-fungus-gardening Termitidae included as outgroup in this study

	**Species name**	**subfamily**
1	*Embiratermes neotenicus*	Syntermitinae
2	*Silvestritermes holmgreni*	Syntermitinae
3	*Cubitermes fungifaber*	Termitinae
4	*Basidentitermes aurivillii*	Termitinae
5	*Microcerotermes biroi*	Termitinae
6	*Neocapritermes taracua*	Termitinae
7	*Drepanotermes sp*	Termitinae
8	*Termes hospes*	Termitinae
9	*Macrognathotermes errator*	Termitinae
10	*Postsubulitermes parviconstrictus*	Nasutitermitinae
11	*Constrictotermes cavifrons*	Nasutitermitinae
12	*Trinervitermes togoensis*	Nasutitermitinae
13	*Trinervitermes trinervoides*	Nasutitermitinae
14	*Trinervitermes geminatus*	Nasutitermitinae
15	*Nasutitermes corniger*	Nasutitermitinae
16	*Anoplotermes Sp*	Apicotermitinae
17	*Compositermes Sp.*	Apicotermitinae
18	*Aparatermes sp.*	Apicotermitinae
19	*Anoplotermes sp.*	Apicotermitinae
20	*Aderitotermes sp.*	Apicotermitinae
21	*Jugositermes tuberculatus*	Apicotermitinae

Table S4 GenBank accession numbers of sequences excluded in this study and reasons of exclusion

Reasons of Exclusion	GenBank Accession Number(s)
**Pseudo COI**	GU254070
**Messy/ambiguous**	FJ207444, FJ207440, FJ207438, FJ207436, FJ207446, FJ207448, AY127748
**Identical**	AB909011, AB909015, AB909013, AB909014, AY127712, JF923294, JF923320, JF923327, JF923331, JF923305, AY818087, EF206316, JF923326, EU253850, EU253847, JQ412138, AB626146, AB626147, KJ887191, KC887191, KJ918309, KJ918340, KJ918341, FJ207435, FJ207449, JF302837, EU253856, FJ802752, JX050221, JF923338, JF923282, JF923301, GU245157, GU254149, GU254144, GU254147, KF430189, KP769535, FJ207431, GQ922734
